# The crucial roles and research advances of cGAS‑STING pathway in liver diseases

**DOI:** 10.1080/07853890.2024.2394588

**Published:** 2024-08-25

**Authors:** Xiaoqian Zhang, Bin He, Juan Lu, Qiongling Bao, Jie Wang, Yida Yang

**Affiliations:** a State Key Laboratory for Diagnosis and Treatment of Infectious Diseases, National Clinical Research Centre for Infectious Diseases, Collaborative Innovation Center for Diagnosis and Treatment of Infectious Diseases, The First Affiliated Hospital, Zhejiang University School of Medicine, Hangzhou, China; b Department of Hepatobiliary and Pancreatic Surgery, The First Affiliated Hospital, Zhejiang University School of Medicine, Hangzhou, China

**Keywords:** cGAS-STING, liver inflammation, innate immunity, mechanism, inhibitors

## Abstract

Inflammation responses have identified as a key mediator of in various liver diseases with high morbidity and mortality. cGAS-STING signalling is essential in innate immunity since it triggers release of type I interferons and various of proinflammatory cytokines. The potential connection between cGAS-STING pathway and liver inflammatory diseases has recently been reported widely. In our review, the impact of cGAS-STING on liver inflammation and regulatory mechanism are summarized. Furthermore, many inhibitors of cGAS-STING signalling as promising agents to cure liver inflammation are also explored in detail. A comprehensive knowledge of molecular mechanisms of cGAS-STING signalling in liver inflammation is vital for exploring novel treatments and providing recommendations and perspectives for future utilization.

## Introduction

The incidence of liver disease is increasingly globally, with approximately two million deaths attributed to it [1]. The liver is the largest solid organ and is prone to various types of injuries and destruction. It is involved in many different physiological activities such as detoxification, metabolism and protein synthesis [2,3]. Inflammation is vital for the development of liver diseases [4]. The aetiology of chronic liver diseases can be viral or toxic, for example, alcoholic or non-alcoholic liver diseases. Ischaemia-reperfusion (IR) damage following liver transplantation and acute liver failure with extensive necrosis also cause inflammation [5]. Hepatocyte death induces the release of damage-associated molecular patterns (DAMPs), which amplify the inflammatory responses in liver and are identified by the innate immune system *via* pattern recognition receptors [6].

Over the past decade, cyclic GMP–AMP synthase (cGAS)-stimulator of interferon gene (STING) signalling has been comprehensively studied. cGAS-STING signalling is involved in various diseases, such as cancer, autoimmune diseases, infections, inflammation and metabolic abnormalities [7]. Recently, cGAS-STING signalling has been implicated in inflammatory disorders. Moreover, although cGAS-STING signalling affects liver injury, hepatocellular carcinoma (HCC), non-alcoholic fatty liver disease (NAFLD) and viral hepatitis [8], the detailed mechanism of cGAS-STING signalling in liver inflammation remains unexplored.

In this article, we summarize the impact of cGAS-STING signalling on various liver inflammatory diseases and evaluate its potential as a therapeutic target for liver inflammation.

## Regulatory mechanisms of cGAS-STING signalling

**Figure 1.**
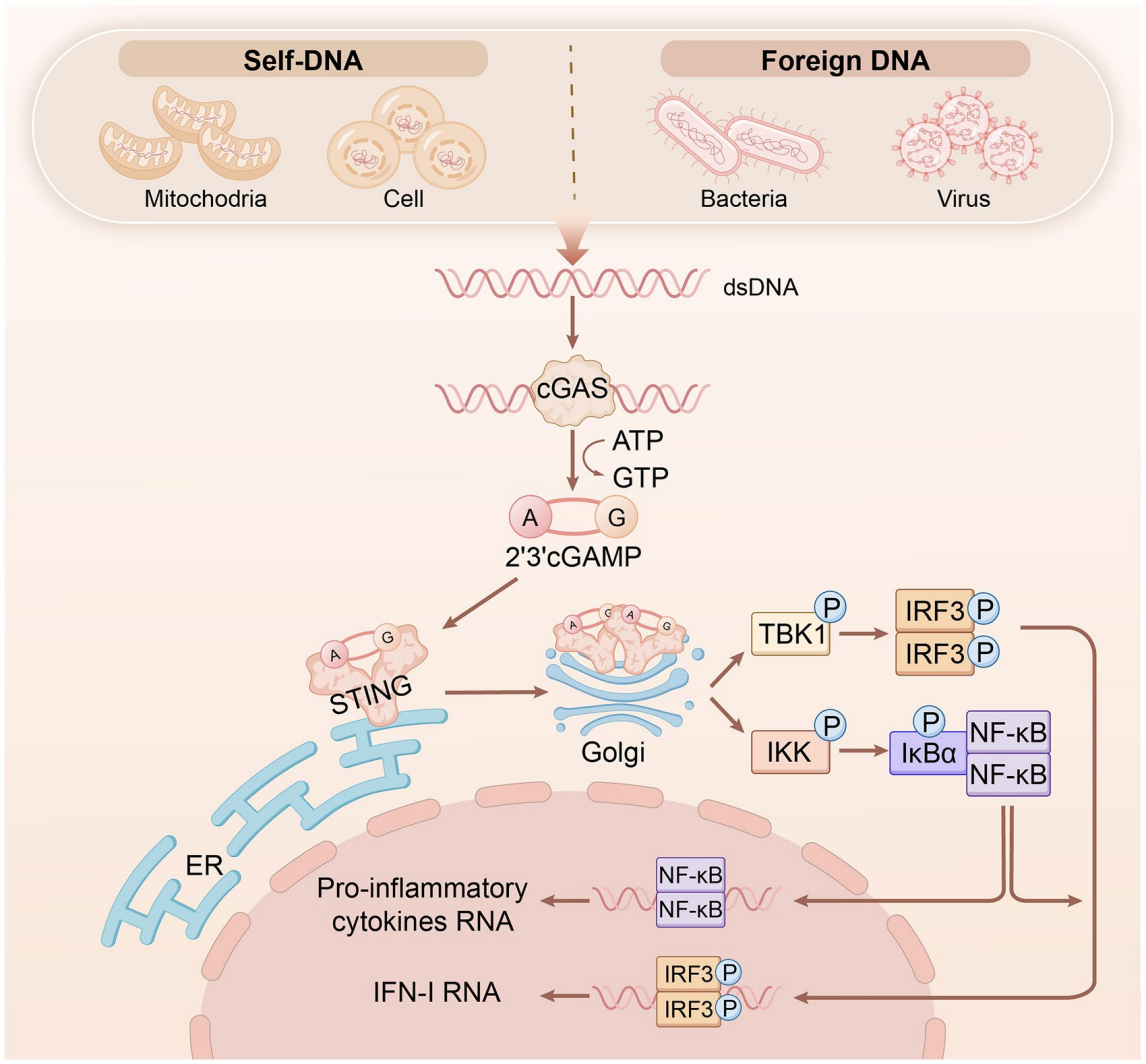
Molecular mechanism of cGAS-STING signalling. The cGAS-STING pathway is stimulated by DNA to against pathogens and induce inflammatory responses. in detail, cGAS senses aberrant or mislocalized dsDNA to generate cGAMP, binding to STING to induce the relocation of STING to Golgi apparatus and trigger activation of TBK1 to result in phosphorylation of both STING and IRF3 transcription factor. In addition, IFN-I is initiated upon translocation of IRF3 into the nucleus, which subsequently induces various IFN-stimulated genes. At the same time, STING has ability to enlist IKK, which then triggers phosphorylation of the NF-kB inhibitor, IkBa, speeding up movement of NF-kB into the nucleus, which in turn enhances the production of certain inflammatory cytokines. cGAS, cyclic GMP-AMP synthase; STING, stimulator of interferon genes; dsDNA, double-stranded DNA; cGAMP, cyclic AMP-GMP; TBK1, TANK-binding kinase 1; IRF3, interferon regulatory factor 3; IFN, interferon; IKK, IkB kinase; NF-kB, nuclear factor-kB.

The cGAS-STING pathway detects DNA that induces potent innate immune defense system [9]. In the ­cytosol, cGAS senses aberrant or mislocalized double-stranded DNA (dsDNA) to generate cyclic AMP-GMP (cGAMP), which stimulates the protein STING [10], and phosphorylates downstream kinases and transcription factors to increase the synthesis of interferons and cytokines, ultimately causing cell death [11–13]. In addition to dsDNA, cytoplasmic manganese (Mn^2 +^) benefit cGAS to sense dsDNA more sensitive during viral infection [14]. And cytoplasmic Mn ^2 +^ can induce cGAS activation and cGAMP production thus to initiate IFN-I responses in the absent of dsDNA without infection [15]. As shown in Figure 1, cGAMP binding to STING induces its relocation to the Golgi apparatus and triggers the activation of TANK-binding kinase 1 (TBK1), leading to the phosphorylation of both STING and Interferon regulatory factor 3 (IRF3) [6]. Type I interferons (IFNs) are activated upon the translocation of IRF3 into the nucleus, which subsequently induces various IFN-stimulated genes [12]. In addition, STING has the ability to enlist IκB kinase (IKK), which then triggers phosphorylation of the nuclear factor-κB (NF-κB) inhibitor, IκBa, accelerating the movement of NF-κB into the nucleus, which, in turn, enhances the production of certain inflammatory cytokines [7].

The cGAS-STING pathway affects autophagy, senescence, and antitumor immunity, and overactivation of this pathway results in inflammatory and autoimmune disorders [10]. In the liver, the cGAS self-DNA sensor is effective in detecting cellular or tissue damage. However, dysregulation of the cGAS-STING pathway leads to inflammation and subsequent illnesses. Thus, the cGAS-STING pathway plays a dual role—temporary stimulation of this pathway has beneficial effects in managing tumours and viruses, whereas continuous stimulation contributes to inflammation-induced tumour formation [16].

### cGAS-STING signalling in immune regulation

**Figure 2.**
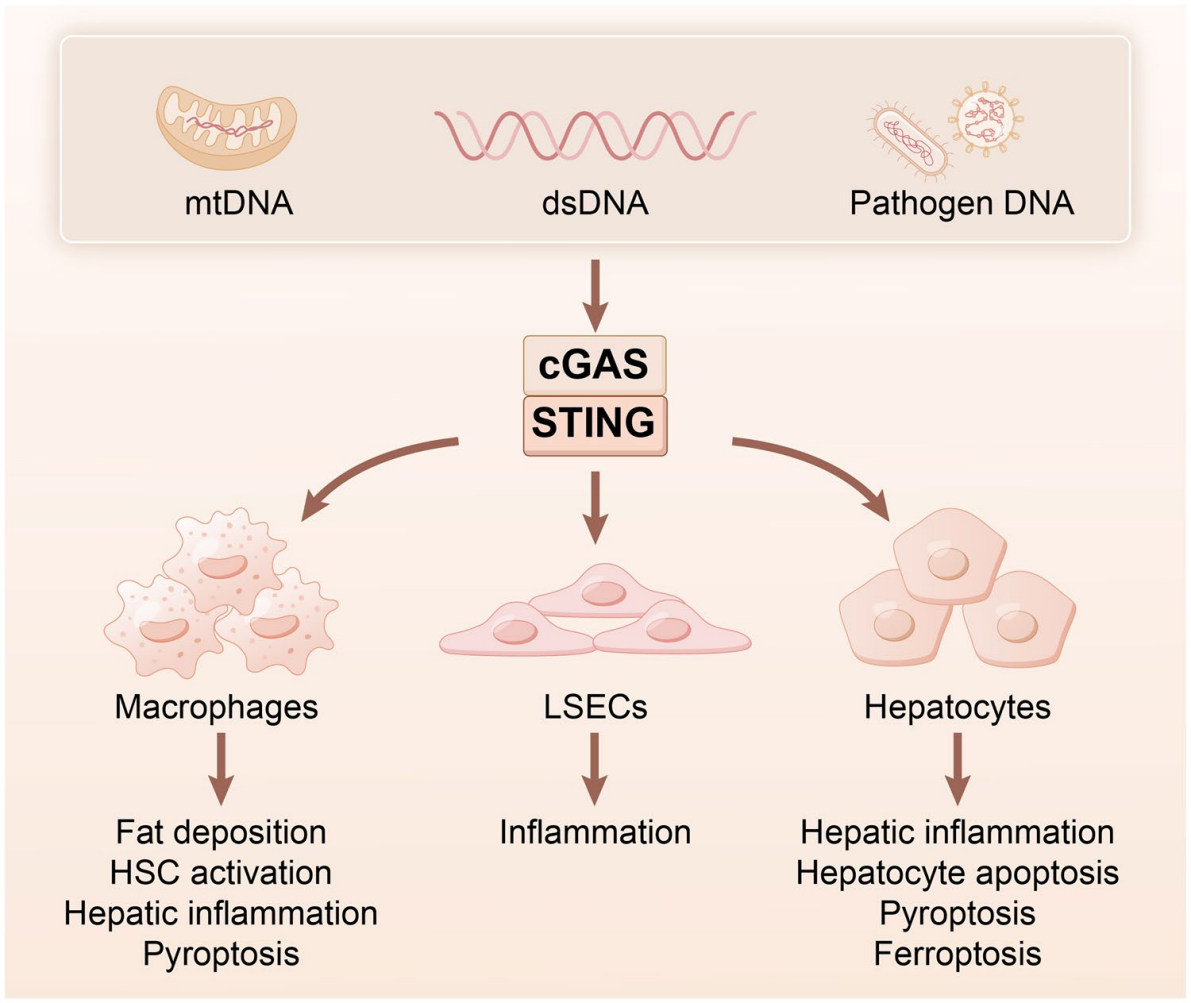
The cGAS-STING signalling during immune response. Both cytosolic and extracellular DNA can activate cGAS-STING and induce innate immune responses in various cell types to led to a range of cellular immune functions. cGAS, cyclic GMP-AMP synthase; STING, stimulator of interferon genes.

Pathogen-associated molecular patterns (PAMPs) and DAMPs are induced by various factors such as DNA disruption, mitochondrial damage, apoptosis, exosomes, DNA viruses, retroviruses and microbes. Both cytosolic and extracellular DNA can act as PAMPs to activate DNA sensors and induce innate immune responses in eukaryotic organisms [17]. Foreign DNA plays a vital role in the immune response of several organisms. In mammalian cells, the cGAS-STING pathway is central to the production of effective innate immune responses *via* DNA [18]. As shown in Figure 2, cGAS-STING signalling is unique among the innate immunity signalling systems because it is triggered by DNA. Consequently, its activation does not depend on the unique properties of pathogens [19]. Therefore, cGAS can detect a wide range of DNA molecules, whether they originate from external sources or from within the organism. Dysregulation of this adaptable innate immune sensing system can disturb the balance between cells and organisms, leading to abnormal innate immune responses that are linked to many diseases [18]. Factors that determine whether a host is effective in preventing infection are still being ­discovered. However, the strength and duration of cGAS-STING is vital in most situations [9].

STING is predominantly expressed and activated in hepatic non-parenchymal cells (NPCs), including Kupffer cells, liver sinusoidal endothelial cells (LSECs), and hepatic stellate cells (HSCs), which coordinate the activation of the immune response when danger signals from pathogens or injured cells and tissues trigger liver inflammation [20]. In addition, cGAS maintains its stability through conformational changes in DNA-bound structures, suggesting that cGAS detects mitochondrial DNA (mtDNA) as a signal that activates the natural immune system, generating cGAMP and a range of cellular immune functions [21].

### cGAS-STING signalling in metabolism

Metabolic reprogramming in macrophages occurs at two branches of the TCA cycle, the cis-aconitic acid efflux point to increase succinate activity of ACDO 1 and inhibition of isocitratedehydrogenase (IDH) to allow conversion of cis-aconitic acid to itaconic acid [22]. The itaconic acid inhibits the activation of STING *via* Nrf 2 [23]. In addition, itaconic acid acts as an inhibitor of succinate dehydrogenase, inhibiting the conversion of succinate to fumaric acid [24]. This prevents glycolysis, the main energy metabolism in M1 *via* HIF-α, which is less efficient than OXPHOS in M2 [25]. Activation of STING-IFN in M2 leads to its reprogramming in M1, a process that can be inhibited by loss of pyruvatedehydrogenasekinase2/4 (PDK 2/4). This also addresses obesity-related insulin resistance [26].

#### Lipid metabolism

**Figure 3.**
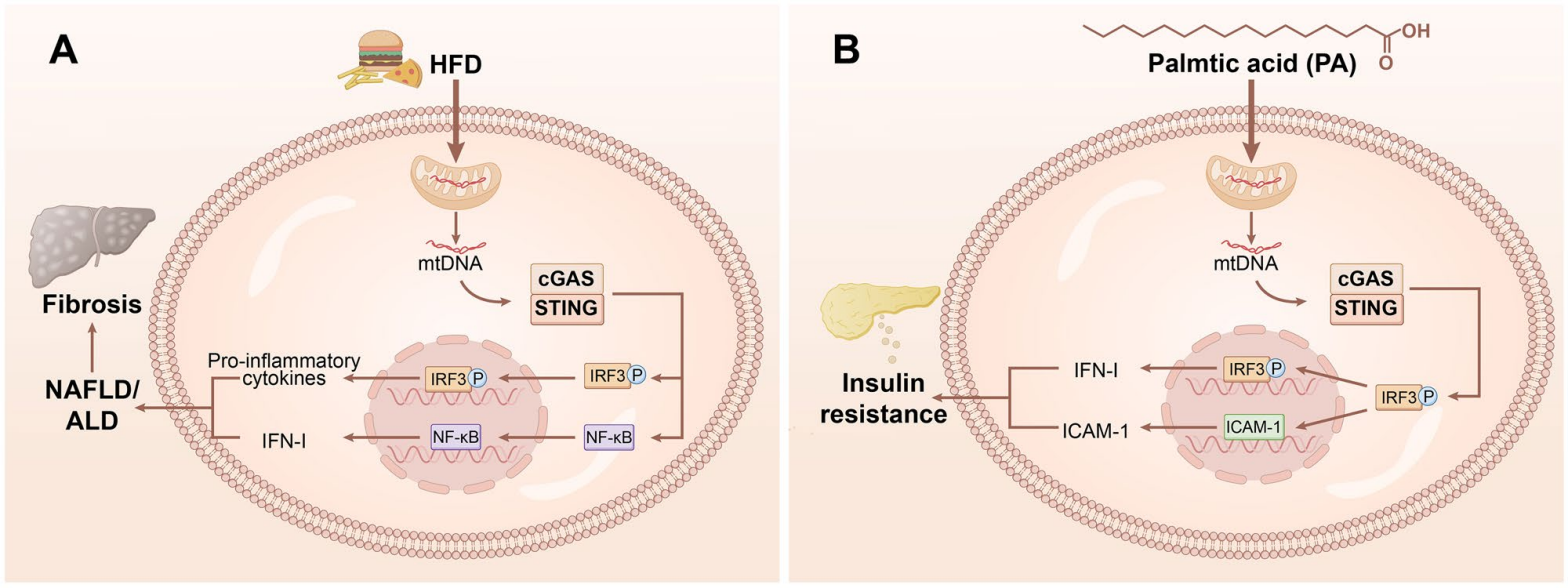
cGAS-STING signalling in metabolism. (A) The cGAS-STING pathway in the liver mediates NAFLD/ALD/fibrosis. HFD/free fatty acids (FFAs) led to mitochondria stress to induce the release of mtDNA. The mtDNA activate cGAS-STING to stimulate IRF3 and NF-KB thus to aggravate proinflammatory cytokines and IFN-I production. The growing inflammation and lipid accumulation together led to NAFLD/ALD and even liver fibrosis. (B) The cGAS-STING pathway mediates glucose homeostasis. Palmitic acid induced mtDNA leakage stimulate cGAS-STING, leading to production of IFN-I and expression of ICAM-1 to induce insulin resistance thus to regulate glucose homeostasis. cGAS, cyclic GMP-AMP synthase; STING, stimulator of interferon genes; NAFLD, nonalcoholic fatty liver disease; ALD, alcoholic liver disease; HFD, high fat diet; mtDNA, mitochondrial DNA; IRF3, interferon regulatory factor 3; NF-kB, nuclear factor-kB; IFN-I, interferon-I.

The cGAS-STING pathway is involved in lipid metabolism (Figure 3(A)); however, the underlying mechanism remains unknown. Evidence indicates that STING activation contributes to fat accumulation by regulating lipid metabolism [20]. Lipotoxicity induces mtDNA, lipid antigens, and adipokines, and activates the cGAS-STING pathway to regulate aberrant lipid metabolism [27]. A study conducted on Drosophila has demonstrated that STING is involved in lipid metabolism [28]. We confirmed the interaction between STING and lipid synthases ACC and FASN in Drosophila. This finding suggests that these three proteins are components of a complex comprising several enzymes. The elimination of STING in Drosophila leads to disrupted positioning of the ACC, decreased FASN enzyme activity, heightened susceptibility to hunger and oxidative stress, diminished lipid storage, and downregulated expression of genes involved in lipid metabolism [28]. Liver X receptor (LXR) agonists have been identified as inhibitors of STING signaling that induce lipid metabolism. SMPDL3A is a cGAMP-specific nuclease, and LXR-associated lipid metabolism stimulates SMPDL3A expression and accelerates cGAMP degradation, thereby inhibiting STING-mediated innate immunity [29,30]. It is revealed that STING proteins regulate metabolic homeostasis by inhibiting fatty acid desaturase 2 (FADS 2) rate-limiting enzymes to suppress the level of polyunsaturated fatty acid (PUFA) desaturation. And PUFA inhibits STING to benefit STING-related inflammation from regulating antiviral response. The negative regulatory feedback loop between STING and FADS-2 modulates the inflammatory response, implying crucial role of metabolic alterations in abnormal STING activation and STING-targeted therapy [31]. These studies highlight the functional link between cGAS-STING and lipid metabolism, and we expect cGAS-STING-related ligands to be a promising therapeutic strategy in the future.

#### Glucose homeostasis

The cGAS-STING plays a role in the insulin signalling pathway to regulate glucose homeostasis (Figure 3(B)). Impaired glucose homeostasis could induce various metabolic diseases, such as obesity, insulin resistance and hyperglycaemia [32]. Endothelial inflammation and activation would be stimulated under theses metabolic stress and endothelial inflammation seems to be an interesting target during glucose homeostasis. The STING pathway induces an inflammatory response in macrophages when confronted with obesity. In detail, STING-IRF3-IFN I signalling led to the balance of biosynthesis and import of lipids by decreasing the lipid synthesis rate and increasing the amount of cholesterol and long-chain fatty acids imported into macrophages [33]. And inhibition of TBK1 reduces macrophage infiltration as well as the level of mRNA encoding key inflammatory genes in adipose tissue, while increasing brown adipose tissue and the rate of fat oxidation and energy consumption [34]. These studies indicate crucial roles for STING-IRF3-TBK1 in regulating the adipocyte infiltration, and macrophage polarization in the context of obesity. In addition, activation of STING-IRF3, highly induced in adipocytes of obese mice and humans, led to insulin resistance in adipocytes [35]. And ablation of IRF3 in mice reduced HFD-induced macrophage infiltration into fat pads, inflammatory gene expression, and insulin resistance [35]. Palmitic acid induces mitochondrial damage and mtDNA into the cytosol to activate STING-IRF3 pathway and to promote the level of ICAM-1 further to aggravate insulin resistance, obesity and glucose intolerance [36], implying STING as a candidate for insulin resistance.

### cGAS-STING signalling in cell death

**Figure 4.**
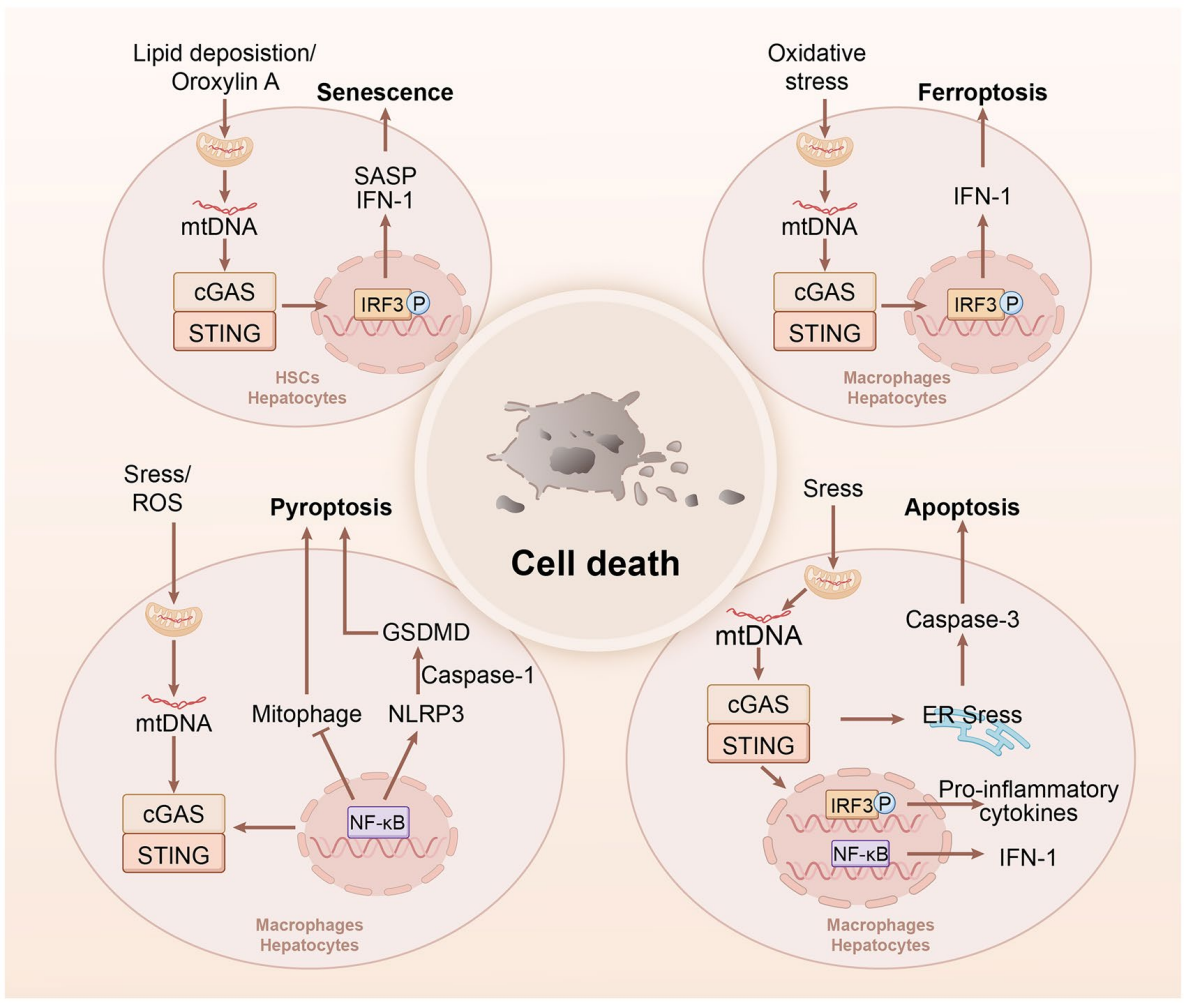
cGAS-STING signalling in cell death. (A) The cGAS–STING pathway in senescence. Lipid deposition/Oroxylin a stimulate the leakage of mtDNA to initiate IFN-I production and SASP *via* the cGAS–STING pathway, resulting cellular senescence. (B)The cGAS–STING pathway in apoptosis. Mitochondrial stress induce the release of mtDNA to activate the cGAS–STING pathway, resulting in IFN-I and cytokines production, which in turn aggravate caspase-3 to promote apoptosis. (C) The cGAS–STING pathway in pyroptosis. Stress/ROS induce mtDNA to activate the cGAS–STING pathway to promote NF-KB signalling, which triggers NLRP3 inflammasome and caspase-1 to activate GSDMD-dependent pyroptosis. In addition, NF-KB can also suppress mitophage to promote pyroptosis. (D) The cGAS–STING pathway in ferroptosis. Stress/ROS induce mtDNA to activate the cGAS–STING pathway to promote IRF3 signalling, which triggers IFN-I production to activate ferroptosis. cGAS, cyclic GMP-AMP synthase; STING, stimulator of interferon genes; mtDNA, mitochondrial DNA; IFN-I, interferon-I; SASP, senescence-associated secretory phenotype; ROS, reactive oxygen species; NF-kB, nuclear factor-kB; NLRP3, nucleotide-binding domain and leucine-rich repeat containing protein 3.

Cell death is a fundamental physiological process in all living organisms that serves both physiological and pathological roles. Unexpected and uncontrolled destruction of cells leads to a significant discharge of cellular components into the extracellular space. These discharged substances function as signals of harm, referred to as DAMPs, to prompt defensive responses from the immune system and attract immune cells and phagocytes to eliminate potential threats and support tissue healing. When infections occur, PAMPs initiate specific immune responses to combat infection [37]. Here we summarize the cGAS-STING signalling in various types of cell death (Figure 4).

Cellular senescence, a form of programmed cell death (PCD), is a condition in which cell proliferation is permanently halted because of prolonged DNA damage and other stress-related signals [38]. Recent research has highlighted several additional roles of senescence, which involve the production of assorted inflammatory agents such as chemokines, growth factors, and interleukins, collectively recognized as the senescence-associated secretory phenotype (SASP) [38,39]. DNA damage response influences cellular senescence, in which SASP is a vital component [40]. The cGAS-STING pathway can activate the SASP and generate DNA fragments within senescent cells. Evidence indicates that curcumol prevents lipid deposition in liver injury by suppressing cellular senescence. Curcumol inactivates the cGAS-STING pathway to reduce SASP-related inflammatory factor secretion and ethanol-induced formation of CCF preventing the combination of LC3B with lamin B1 from affecting nuclear membrane integrity. This suggests that curcumol alleviates AFLD by inhibiting the SASP-cGAS-STING pathway [41]. Oroxylin A induces HSC senescence during liver fibrosis, both *in vitro* and *in vivo*, by activating the cGAS-STING pathway, which is mainly offset by DNMT3A overexpression [42]. Furthermore, oroxylin A therapy relieves the abnormal alterations in liver fibrosis, decreases collagen accumulation, and effectively suppresses liver fibrosis by activating HSC ferritinophagy, and further inducing HSC senescence. Oroxylin A stimulates cGAS-STING pathway to enhance the secretion of cytokines such as IFNβ, leading to upregulation of NCOA4 and subsequent regulation of ferritinophagy, indicating that the cGAS-STING pathway regulation of HSC senescence is a potential target for liver fibrosis therapy [43]. Reduced YAP/TAZ mechanotransduction contributes to senescence by activating cGAS-STING signalling. YAP/TAZ activity decreases in stromal cells during normal ageing and replicating this decrease by genetically deactivating YAP/TAZ in these cells results in rapid ageing. Disabling YAP/TAZ leads to the development of ageing characteristics that are preceded by the activation of tissue senescence. YAP/TAZ regulates cGAS-STING signalling by maintaining nuclear envelope integrity through the direct transcriptional control of lamin B1 and ACTR2, which are essential for the formation of the perinuclear actin cap. Therefore, maintaining YAP/TAZ mechanosignalling or blocking STING could be effective strategies for reducing senescence-related inflammation and enhance healthy senescence [44].

Apoptosis is a form of PCD that releases cytochrome c from the mitochondria and is controlled by the equilibrium of anti-apoptotic and pro-apoptotic proteins in the BCL-2 family, as well as effector caspases (e.g. caspase-3, -6, and -7) and initiator caspases (e.g. caspase-8, caspase-9 and caspase-10) [37]. This process reaches its peak when caspase-6 fragments the nuclear membrane, cleaving several intracellular proteins (e.g. caspase-3 and PARP) and causing membrane bubbles to integrate DNA into nucleosomal structures [45]. These events represent characteristic signs of apoptosis and are frequently utilized as typical indicators of cell death. During apoptosis, the released mtDNA is sensed by cGAS-STING, stimulating an inflammatory response. The cGAS-STING pathway promotes apoptosis in various liver diseases. Acetaminophen (APAP)-induced mice hepatotoxicity is attributed to inflammatory responses and apoptosis by activation of the cGAS-STING signalling pathway. Emodin prevents APAP-induced liver injury by stimulating the nuclear factor erythroid 2-related factor 2 (Nrf2)-mediated antioxidant stress pathway and suppressing the cGAS-STING pathway to inhibit apoptosis [46]. Wang et al. found that human mesenchymal stem cells (hMSCs) could alleviate liver injury by promoting M2 macrophages to induce stress granules against damage due to stress. hMSCs could decrease reactive oxygen species (ROS) production, apoptosis and endoplasmic reticulum (ER) stress to inhibit the cGAS-STING pathway, reducing TNF-α, IL-6, IL-1β mRNA expression in macrophages, which weakens the inflammatory responses to ameliorate hepatocytes damage [47]. In HCC, STAT3 knockdown can strengthen sorafenib-induced ER stress-induced apoptosis, where delivered DNA induces cGAS-STING pathway in CD103+ dendritic cells (DCs) to induce IFN-I production. Finally, CD8+ T and natural killer cells (NKs) can be induced to improve the anti-HCC immune responses. The enhanced anti-HCC ability of the ­combination of sorafenib with STAT3 knockdown was attributed to apoptosis induced by the DNA-cGAS-STING-type I IFNs axis in DCs [48]. Altogether these studies verify effects of cGAS-STING pathway on apoptosis in liver diseases.

Pyroptosis is a form of cell death that induces inflammasome sensing and blocks plasma membrane integrity. These sensors are of various types, such as the Nod-like receptor family, DNA receptor absent in melanoma 2 (AIM2), and pyrin receptor. These sensors identify various PAMPs and DAMPs emitted by invading microorganisms or disrupted cellular processes. When confronted with central microbial spread and potential threats, inflammasomes of the immune system induce lytic cell death and act as a robust defense against infections or cell stress [37]. cGAS-STING signalling is vital to pyroptosis in various liver diseases. In thioacetamide (TAA)-induced acute liver injury (ALI), hepatocyte-specific XBP1 knockout mice showed worsened ALI with elevated hepatocellular pyroptosis *via* release of mtDNA and activation of the cGAS-STING pathway. These findings indicate that macrophage XBP1 deficiency enhances pyroptosis by inhibiting mitophagy, resulting in activation of mtDNA/cGAS/STING signalling, suggesting a promising role in treatment options for ALI [49]. In mice with hexafluoropropylene oxide trimer acid (HFPO-TA)-induced liver fibrosis, mitochondrial ROS (mtROS) production was increased, inducing cGAS-STING signalling as an upstream regulatory mechanism of pyroptosis and fibrosis. HFPO-TA promotes liver fibrosis through mtROS/cGAS-STING/NLRP3-induced pyroptosis [50].

Ferroptosis, a distinct form of cell death influenced by iron-dependent phospholipid peroxidation and discovered in 2012, has been controlled by several cellular metabolic pathways such as redox balance, iron regulation, mitochondrial function, and amino acid, lipid, and sugar metabolism, along with different signalling pathways associated with diseases. Ferroptosis is involved in the regulation of organ damage and degenerative diseases [51]. Accumulating evidence indicates that ferroptosis influences cGAS-STING signalling in liver diseases, suggesting that oxidative stress-induced ferroptosis and macrophage-associated inflammation are important factors in several liver disorders. Su et al. showed that hepatocyte-specific TAK1-deficient mice exhibit notable liver damage and elevated intrahepatic M1 macrophage counts. Ferrostatin-1, an inhibitor of ferroptosis, alleviates liver damage and fibrosis, and decreases tumour burden, which in turn blocks the activation of macrophage STING signalling. Oxidative DNA damage resulting from hepatocellular ferroptosis activates STING signalling in macrophages, leading to liver injury, fibrosis, and cancer. Therefore, blocking macrophage STING signalling may be a novel treatment strategy for the prevention of chronic liver disease [52]. Li et al. established that the ginsenoside Rd ameliorated ALI in mice with reduced amounts of serum and liver iron, 4-hydroxynonenal, and 8-hydroxy-2 deoxyguanosine, as well as decreased expression of cGAS and STING. Erastin, a ferroptosis inducer, effectively counteracted the hepatoprotective effects and impact of ginsenoside Rd on specific markers, indicating that ginsenoside Rd prevents ferroptosis by inhibiting the cGAS-STING pathway, thereby shielding mice against carbon tetrachloride (CCl4)-induced ALI [53]. Manganese activates cGAS-STING to promote mitochondrial lipid peroxidation and ROS production by releasing type I IFN to reduce DHODH function, thereby inducing ferroptosis in tumour, providing a new strategy to complement existing anti-tumour treatment options [54]. On the other hand, cGAS is found to protect HCC from ferroptosis. cGAS localizes in mitochondrial membrane to form oligomerization with dynamin-related protein 1 to inhibit ferroptosis, indicating the potential role of cGAS in regulating ferroptosis and novel targets for cancer [55].

## The cGAS-STING signalling pathway in inflammatory liver diseases

### Viral hepatitis

**Figure 5.**
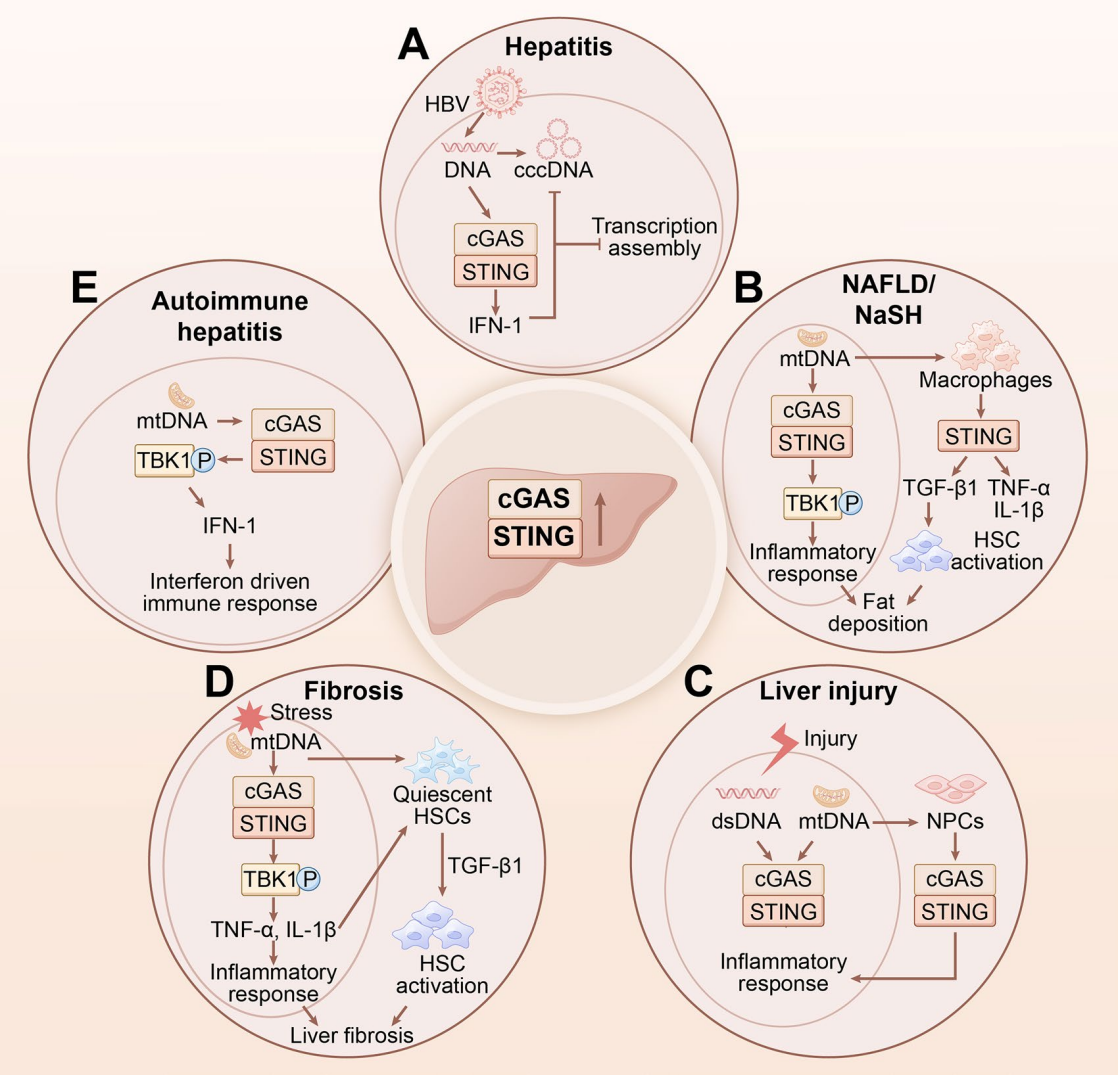
cGAS-STING signalling in liver inflammatory diseases. (A) Stimulation of cGAS-STING signalling result in inhibition of HBV replication. (B) Mitochondrial DNA induces cGAS-STING-TBK1 to activate liver inflammations to aggravate fat deposition. (C) Liver injury varies from different forms activate cGAS-STING signalling to induce inflammatory responses. (D) When confronted with stress, released mitochondrial DNA stimulates cGAS-STING signalling to exacerbate inflammations and HSC activation finally to resluting in fibrosis. (E) Mitochondrial DNA induces cGAS-STING-TBK1 to IFN-I driven immune responses to lead in autoimmune hepatitis. cGAS, cyclic GMP-AMP synthase; STING, stimulator of interferon genes; HBV, Hepatitis B Virus; TBK1, TANK-binding kinase 1; HSC, hepatic stellate cell; IFN-I, interferon-I.

Globally, viral hepatitis is a serious public health problem with high morbidity and mortality rates. Hepatitis B Virus (HBV) infection remains the leading cause of chronic hepatitis, liver cirrhosis, and HCC [56]. The innate immune response is activated during viral replication when host factors recognize viral replication intermediates. As a critical cytosolic DNA sensor, cGAS-STING has drawn considerable research attention owing to its antiviral capability and role in innate immunity [57–59] and many studies have focused on viral hepatitis (Figure 5(A)).

HBV interacts with the signalling pathways of hepatocyte innate immunity and suppresses its function. Hence, it is crucial to explore the interactions between HBV and innate immunity to identify novel therapeutic approaches for treating HBV infections. Stimulation of cGAS-STING signalling may considerably inhibit HBV replication *in vitro* and *in vivo*. Conversely, blocking cGAS promotes HBV DNA accumulation [60]. Mechanistically, cGAS-STING signalling is responsible for innate immune responses to HBV and preventing HBV assembly [61]. The hepatitis B virus X protein (HBx) is a crucial regulatory protein in HBV. It acts as an antagonist of the cGAS-STING pathway. HBx inhibits the release of type I IFN by directly facilitating the ubiquitination and autophagy destruction of cGAS, which inhibit the host cGAS DNA-sensing mechanism to enhance the replication of HBV [62]. Therefore, the cGAS-STING pathway is involved in the monitoring of HBV infection and could be utilized for the development of innovative anti-HBV approaches. For instance, Schisandra Chinensis demonstrates antiviral activity against HBV, resulting in decreased levels of HBeAg, HBcAg, HBsAg and HBV DNA; Schisandra Chinensis activates cGAS-STING signalling to promote the expression of TBK1, critical for IRF3 phosphorylation and IFNβ generation [63]. Shu et al. examined the effect of RVX-208, a small molecule that enhances the expression of apoA-I, on the inhibition of HBV. RVX-208 stimulates the cGAS-STING pathway to induce cytokines with antiviral properties such as IFNs, proinflammatory cytokines, and chemokines [64]. However, the processes controlling cGAS stability, particularly feedback regulation during viral infections, remain unclarified. In mice peritoneal macrophages, viral infection triggers the activation of the UAF1-USP1 deubiquitinase complex, which specifically combines with cGAS to cleave its K48-linked polyubiquitination, enhancing its protein level and cGAS-dependent type I IFN responses. Antiviral responses induced by cGAS are inhibited by a deficiency in Uaf1 and inhibitors of the UAF1-USP1 deubiquitinase complex, thus promoting viral infection [65].

### Liver injury

The liver can be damaged by various factors, including alcohol consumption, medications, radiation and IR. Hepatocyte necrosis or apoptosis caused by injury leads to the release of nuclear DNA or mitochondrial DNA (mtDNA). These molecules act as DAMPs and induce innate immune responses, causing sterile liver inflammation [66]. As a DNA sensor, cGAS has been reported to be activated and stimulate STING in various types of liver injuries (Figure 5(B)).

#### Drug-induced liver injury (DILI)

DILI is defined as damage due to the use of suspected drugs. DILI can be classified into hepatocellular, cholestatic, or mixed types and may also involve immunological reactions [67]. APAP is a widely used analgesic and antipyretic medication, and both intentional and inadvertent overdose may result in severe nephrotoxicity and hepatotoxicity, inducing acute liver failure and kidney injury [68,69]. The primary mechanisms underlying APAP-induced liver failure are inflammatory responses and oxidative stress [70]. APAP-induced liver injury shows increased cGAS-STING signalling. APAP suppresses Nrf2-mediated antioxidative stress and stimulates the NLRP3 inflammasome by inducing the cGAS-STING signalling pathway, thus accelerating liver injury [46]. In liver NPCs, APAP-mediated necrosis contributes to DNA release and activates cGAS-STING signalling, leading to further type 1 IFN production, which amplifies liver injury [71]. cGAS-STING boosts inflammatory factors, further inhibiting M2-type macrophages to promote inflammatory responses in APAP-treated mice, resulting in hepatocyte damage [47].

#### Liver IR injury

Liver IR injuries are the main cause of hepatic dysfunction and failure. Ischemia-induced mtDNA activates macrophage cGAS-STING to promote liver IR injury through an elevated NLRP3-mediated inflammatory response in aged livers [72,73]. A recent study demonstrated that STING triggers liver IR injury by facilitating calcium-dependent caspase 1-GSDMD processing in macrophages and that STING knockdown reduces liver IR injury [74]. Activation of the STING pathway is involved in liver IR injury. MiR-24-3p has been reported to reduce cell death and ameliorate liver inflammatory responses during hepatic IR, possibly through a mechanism inhibiting STING [75], representing a potential therapeutic approach in the clinic. In addition, the cGAS-STING pathway activated by Sirt3 induces hepatocyte death through cytosolic mtDNA releasing to result in cGAS transcription liver IR injury model [76], indicating cGAS-STING a promising target in liver IRI.

#### Radiation-induced liver injury

Radiation-induced liver injury is associated with a high mortality rate [77]. The dsDNA released after irradiation rapidly triggers the cGAS-STING pathway in NPCs, resulting in the synthesis and release of IFN-I and simultaneous damage to hepatocytes [8].

#### Sepsis-associated acute liver injury

Similarly, sepsis-associated acute liver injury contributes to the release of large amounts of dsDNA. cGAS-STING signalling is activated during sepsis-induced liver injury and cGAS deficiency in mice significantly attenuates liver injury, liver dysfunction by inhibiting IFN-I responses and hepatocyte death [78]. Furthermore, exosomes derived from bone marrow mesenchymal stem cells could markedly alleviate septic liver injury by inhibiting cGAS-STING signalling, amplifying mitophagy, and decreasing the release of mtDNA into the cytosol [79]. Thus, cGAS-STING signalling plays an important role in septic liver injury and is a potential target for its treatment.

## Non-alcoholic fatty liver disease (NAFLD), non-alcoholic steatohepatitis (NASH), and alcoholic liver disease (AFLD)

STING expression has been found to be significantly upregulated in liver of patients with NAFLD [20]. In NAFLD patients, STING activation is associated with development of liver inflammation and fibrosis by monocyte-derived macrophages, indicating that STING contributes to the progression of NAFLD (Figure 5(C)). The number of STING-positive cells in the liver tissues of patients with NAFLD increases with inflammation and fibrosis, suggesting that STING influences NAFLD progression and may be a key regulator of inflammation and fibrosis [80]. Researchers found that NAFLD hepatocytes rapidly increase in number and have widespread disturbances in DNA replication, characterized by a slowdown in the pace of replication forks and activation of the ataxia telangiectasia and Rad3-related (ATR)/CHK1 pathways. Therefore, DNA damage related to replication builds up in hepatocytes affected by NAFLD, triggering activation of the cGAS-STING pathway to connect replication stress to the IFN-I response [81]. Moreover, bacterial DNA is present in the liver tissue of patients with NAFLD, which may be derived from the gut microbiota. The presence of these bacteria correlates with the severity of NAFLD and development of liver fibrosis. Mechanistically, bacterial extracellular vesicles (bEVs) released by the gut microbiota induce inflammation and fibrosis in NAFLD by activating cGAS-STING signalling, suggesting that the gut microbiota and bEVs are crucial for the pathogenesis and development of NAFLD and are new targets for the treatment of NAFLD [82].

Inflammation and fibrosis are common manifestations of NASH [83]. Hepatic steatosis is characterized by the accumulation of substantial quantities of triglycerides in the hepatocytes. Prolonged fatty liver can lead to hepatocyte death, which can progress to NASH, liver cirrhosis, and HCC [84]. p62 inclusions in hepatocytes serve as a crucial indicator to differentiate between simple fatty liver disease and NASH and indicate an unfavourable prognosis for HCC. Mechanistically, cGAS and STING are upstream regulators responsible for activating the lipotoxic properties of TBK1 and phosphorylating p62 to further increase ubiquitin-p62 aggregates [85]. cGAS-STING signalling is involved in the progression of NASH, and modulating this route could potentially serve as a novel treatment approach [20,80]. Licorice is a commonly used herb that is known for its anti-inflammatory and hepatoprotective effects. In mouse NASH models, licorice extract has the ability to hinder cGAS-STING pathway by suppressing STING oligomerization, thus alleviating hepatic fibrosis [86].

AFLD is a persistent liver disease characterized by abnormal accumulation of fat. This condition is mostly caused by long-term heavy alcohol intake [87]. If not treated appropriately, AFLD can eventually lead to development of alcoholic hepatitis, liver fibrosis, liver cirrhosis and HCC [88]. As the potential mechanism of AFLD remains unknown, few efficacious methods can reverse AFLD or impede its progression to more severe conditions. Jin et al. found that curcumol significantly suppressed the release of cytoplasmic chromatin fragments (CCF) produced by ethanol and activated cGAS-STING, impairing the secretion of inflammatory markers associated with SASP [41]. These findings indicate the potential applications of cGAS-STING signalling in AFLD therapies. Alcohol decreases the hepatic expression of DRP1, leading to an increase in mega­mitochondria and mitochondrial maladaptation. Mechanistically, alcohol reduces DRP1 levels by promoting the formation of enlarged mitochondria and inhibiting mitophagy, which leads to liver damage and inflammation caused by elevated levels of cytosolic mtDNA and impairs mitochondrial function, triggering the activation of the cGAS-STING-IFN signalling pathways [89]. This study provides evidence for the crucial involvement of mitochondrial dynamics and mitophagy with cGAS-STING signalling in safeguarding against ALD.

### Liver fibrosis

Liver fibrosis is a chronic disease caused by inflammatory and immunological responses. It is identified as an excessive accumulation of extracellular matrix. Currently, efficient therapeutic approaches are lacking for the management of liver fibrosis, which demonstrates significant mortality globally [90]. Increasing evidence indicates that the cGAS-STING pathway influences liver fibrosis (Figure 5(D)). In the CCl4 mouse liver fibrosis model, TDP-43 expression progressively increases along with activation of cGAS-STING signalling and an increase in inflammatory factors upregulated through the NF-κB pathway. In addition, co-localization of mitochondria and TDP-43 affects the severity of liver fibrosis. These results suggested that TDP-43 influences liver fibrosis and aggravates inflammation by upregulating the cGAS-STING pathway [91]. These findings indicate that CCl4 stimulated the release of mtDNA and significantly enhanced the immunological response mediated by cGAS-STING. This effect was suppressed by MitoQ, which effectively prevented the development of liver fibrosis, demonstrating that mitochondrial oxidative stress is involved in the development of liver fibrosis induced by CCl4. Hence, mitigating or reversing mitochondrial damage could be a promising strategy for treating liver disorders caused by CCl4 [92]. In mice, Xbp1 deficiency ameliorates liver fibrosis by reducing the cytosolic release of mtDNA owing to oxidative mitochondrial injury to suppress NLPR3 activation in a cGAS-STING IRF3-dependent manner [90]. STING activates NLRP3 inflammatory vesicles through an epigenetic mechanism, which mediates hepatocyte pyroptosis and hepatic inflammation in liver fibrosis. By inhibiting the STING-NLRP3 signalling pathway, liver fibrosis can be attenuated and potential mechanisms involving oxidative stress and metabolic reprogramming can be revealed [93]. In chronic CCl4-induced liver fibrosis in mice, induction of cGAS-STING signalling promotes the dysfunction of LSECs to increase sinusoidal microthrombosis, which results in increased portal vein pressure, thus aggravating the degree of fibrosis [94]. However, the pro-fibrotic or anti-fibrotic effects of cGAS-STING remain contested. HSC senescence attenuates liver fibrosis. Manganese (MN) exhibits significant antifibrotic effects by inducing senescence and eliminating activated HSCs, which rely on activated cGAS-STING signalling to promote prosenescence with enhanced immune clearance [95]. Oroxylin A induces senescence by suppressing cGAS gene methylation to prevent methionine metabolites from stimulating the cGAS-STING pathway, eventually ameliorating fibrosis [42]. Furthermore, oroxylin A induces cGAS-STING pathway to stimulate cytokines such as IFNβ to activate ferritinophagy to prevent liver fibrosis. Importantly, cGAS siRNA partially counteracts this effect [43].

### Autoimmune hepatitis(AIH)

The recognition of IFN-I and self-nucleic acids as key factors during the development of systemic autoimmune diseases has led to significant interest in the role of the cGAS-STING pathway in these conditions [9]. Ventus Therapeutics has advanced VENT-03, the first cGAS-STING pathway inhibitor, into phase I clinical trials, thus initiating a new journey in the treatment of autoimmune diseases using cGAS-STING inhibitors. This trial was designed to evaluate the safety of VENT-03 in healthy volunteers, with subsequent trials planned for the treatment of more complex autoimmune diseases, such as systemic lupus erythematosus (SLE) and systemic sclerosis [96]. Hou et al. revealed that CCDC50, a recently discovered autophagy ­receptor, suppresses STING-directed IFN-I signalling by ­transporting K63-polyubiquitinated STING to the autolysosomes for destruction. CCDC50 downregulation enhances the immunological response mediated by cGAS-STING, which is induced by serum from SLE patients [97], implying that cGAS-STING maybe a possible target for autoimmune diseases.

AIH is a chronic inflammatory disease characterized by continuous autoimmune responses that directly target the liver [98]. However, only a few documented instances exist of cGAS-STING signalling being implicated in autoimmune liver diseases (Figure 5(E)). Mn aggravates liver damage and hepatic inflammation induced by ConA by activating cGAS-STING signalling. Moreover, cGAS knockout alleviates Mn-induced liver damage [99], broadening our perspective for future investigations into the treatment and prediction of AIH. Genetic vulnerability is the main factor implicated in autoimmune liver disease, which causes an imbalance in both humoral and cellular immunity. This imbalance induces the production of autoimmune antibodies and T-cell-mediated autoimmune responses, leading to injury. Because the cGAS-STING pathway is central to both innate and adaptive immunity, it may participate in autoimmune liver disease. Further research is needed to elucidate the effects of cGAS-STING in AIH.

### Clinical applications of cGAS-STING signalling in diseases

**Figure 6.**
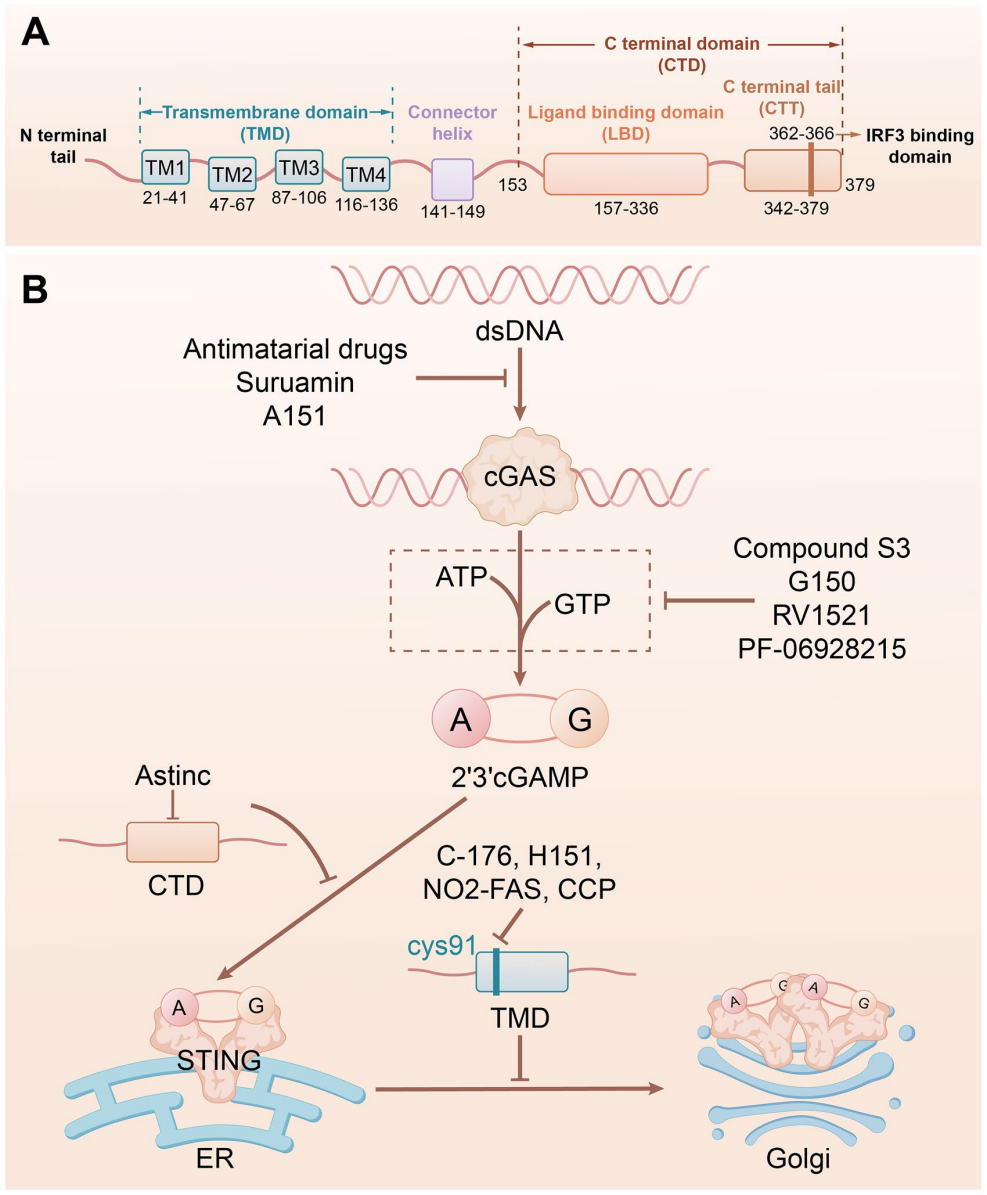
The mechanism of cGAS-STING inhibitors in application. (A) The schematic diagram indicates the functional structural domains of human STING. (B) The schematic diagram shows the mechanisms of inhibitors at different stages during activation of cGAS-STING. cGAS, cyclic GMP-AMP synthase; STING, stimulator of interferon genes.

Studies on the role of cGAS-STING in inflammatory liver diseases with inadequate or unfulfilled treatment options suggest its potential as a target for therapeutic development. The cGAS-STING pathway triggers sterile inflammation, which is crucial for liver damage. Therefore, inhibitors of the cGAS-STING pathway are prospective targets for the treatment of inflammatory liver diseases. Here we summarize the inhibitors in applications in Tables 1 and 2 and the mechanisms in Figure 6.

**Table 1.** cGAS Antagonists used in inflammatory or autoimmune diseases.

Compounds/Molecules	Experimental models	Functions/Biological effects	Refs
PF-06928125	in AGS(Aicardi–Goutières syndrome) mouse models	Occupy the active binding checkpoint of cGAS to compete with cGAMP.	[100]
RU.521	in BMDMs	Occupy the active site of the enzyme to compete with ATP and GTP.	[101]
G150	in primary H-macrophages	Occupy an cGAS catalytic binding pocket similar to that of GTP and ATP.	[102]
Compound S3	in pyrophosphatase (PPiase) -coupled assay	Binds with R376 and N482 at an cGAS catalytic binding pocket.	[103]
Disrupt DNA binding inhibitors (Antimalarial drugs, Suramin, A151)	in THP-1 cells in THP-1 cells in TREX1-deficient cells	They focus on dsDNA-binding domain to disrupting the association between cGAS and dsDNA.	[104–107]
Baicalin and its aglycone baicalein	in pyrophosphatase (PPiase) -coupled assay	They inhibiti cGAS at the molecular level.	[108]

**Table 2.** STING Antagonists used in inflammatory or autoimmune diseases.

Compounds/Molecules	CDNbased (Yes or No)	Experimental models	Functions/Biological effects	Refs
Astin C	Yes	in HEK293 cells in Raw264.7 cells in Trex1^+/-^mice	Targets the CDN sites to diminish the combination of IRF3 and STING signaling, blocking the STING pathway.	[109,110]
c-176	No	in HEK293T cells in Trex1−/− mouse model	Hinders the interaction between STING and CDN by binding to Cys-91 in rat STING, thereby preventing STING palmitoylation	[111]
H151	No	in HEK293T cells in THP-1 cells in Trex1−/−Ifnb1Δβ-luc/Δβ-luc mice	Hinders the interaction between STING and CDN by binding to Cys-91 to prevent palmitoylation of STING	[111]
NO2-FAS	No	in THP-1 cells	Targets the N-terminal domain of STING to induce covalent connections between the Cys88 and Cys91 residues.	[112,113]
CCCP	No	in Raw264.7, HEK293T, HeLa cells	Inhibits and diminishes the phosphorylation of STING to impair the mitochondrial membrane potential to disrupt interaction between TBK-1 and STING.	[114]

### cGAS inhibitors

cGAS functions as a sensor and an enzyme that catalyses the second messenger, cGAMP, for STING signalling. Aberrant cGAS activation is linked to many immune-mediated inflammatory diseases, making it a promising candidate for improving cGAS- and STING-dependent inflammatory disorders.

An important step in the cGAS-STING pathway, cGAS catalyses the conversion of GTP and ATP into 2′,3′-cGAMP upon detecting either internal or external DNA [104,115]. Therefore, limiting the interaction between cGAS and DNA to suppress its enzymatic functions is the viable approach to avoid cGAS-STING pathway.

#### Catalytic site inhibitors

PF-06928125 binds to the active site of cGAS at a location that usually occupies the adenosine base of cGAMP or ATP. Although PF-06928125 bound to cGAS in the biochemical tests, it did not exhibit inhibitory effects in the cellular tests. Blocking the active site may necessitate stronger chemicals, owing to the elevated quantities of ATP and GTP inside the cell [100].

RU.521, using recombinant mouse cGAS (m-cGAS) with a RapidFire mass spectrometry instrument in a high-throughput screening process, is discovered through further chemical synthesis based on structural guidance. It has a crystal structure that is coupled to cGAS with dsDNA located at the active site of cGAS, with the highest potency in a cellular assay. It is widely used in the mouse model of Aicardi–Goutières syndrome to inhibit cGAS activity [101].

G150 is a chemotype G compound with a pyridoindole tricyclic core. It demonstrates no off-target effects on G150 in various experiments assessing inhibition in the innate immune system [102].

Compound S3 exhibits an IC50 of 4.9 µM in PPiase-coupled assay combined with docking assessment. The co-crystal structures of h-cGAS/compound 3 showed that they interacted with the active site of cGAS in a manner consistent with anticipated binding based on simulated screening [103].

#### Inhibitors that disrupt DNA binding

While research groups have also focused on inhibiting cGAS’ active site, an alternative technique could involve disrupting the association between cGAS and dsDNA, which induces cGAS expression.

Antimalarial drugs, like hydroxychloroquine and quinacrine, are being referred to promising therapies for SLE which are known to suppress IFNβ expression through specifically disrupting interaction of cGAS-dsDNA [105].

Suramin has been identified as a cGAS inhibitor that binds to the dsDNA-binding site to suppress the cGAS-dsDNA complex formation. Suppression of cGAS is specific because it does not impact the TLR4 pathways [104].

A151 is an oligodeoxynucleotide that suppresses TLR9 signaling and AIM2 [106]. It inhibits cGAS *via* a competing dsDNA-binding domain. A151 could successfully suppress IFN-I expression in TREX1-deficient cells, suggesting a potential treatment for autoimmune disorders triggered by dsDNA. However, the specific binding site for A151 remains unknown and requires further research for a comprehensive understanding [107].

#### Inhibitors that targeting cGAS

cGAS-specific inhibitors primarily occupy catalytic sites that hinder the formation of cGAMP. Additionally, scaffolds for cGAS inhibitors are scarce, necessitating the discovery of new inhibitors that can accelerate drug development targeting cGAS.

Baicalin and its aglycone baicalein. Among natural flavonoids, baicalin and baicalein are recognized for their potent anti-inflammatory activity. Recently, baicalin and baicalein were identified as inhibitors of cGAS. A further virtual screening process utilizing information from the crystal structures of baicalein revealed a new inhibitor with enhanced effectiveness. Baicalin and baicalein can potentially reduce inflammation by inhibiting cGAS at the molecular level and serve as examples of flavonoids for the discovery of new cGAS inhibitors [108].

### STING inhibitors

Two primary methods are recommended to verify the efficacy of STING inhibitors. One approach involves compounds that bind to cyclic dinucleotide (CDN)-binding sites, effectively blocking STING activation. Another method involves identifying antagonists that focus on either Cys88 or Cys91 residues close to the transmembrane region of STING [9], both of which are palmitoylated.

#### Targeting the CDN-binding site

Tetrahydroisoquinolines. Inhibitors target CDN-binding sites based on the meristic structure of the CDN-binding domain. Compound 1 is a low-affinity agent that competes for the CDN site, resulting in STING inactivation [106].

Astin C, as a natural agent, targets the CDN sites. Mechanistically, Astin C diminishes the combination of IRF3 and STING signaling, blocking the STING pathway [109]. And it shows a potently anti-inflammatory effects in an HSV-1infection model and its therapeutic potential in the treatment of autoimmune diseases [110].

#### Targeting STING palmitoylation site

c-176, as a type of nitrofuran ramification, hinders the interaction between STING and CDN by binding to Cys-91 in rat STING, thereby preventing STING palmitoylation [111].

H151 can inhibit human STING similarly to c-176 [111].

Nitro-fatty acids (NO2-FAS), targeting the N-terminal domain of STING could induce covalent connections between the Cys88 and Cys91 residues. This process suppresses palmitoylation of STING, ultimately inducing inactivation [112]. NO2-FAs suppresses STING palmitoylation in SAVI patient-derived fibroblasts [111]. And NO2-FA treatment also decreases inflammatory markers in heart tissues in *Trex1*^-/-^ model [113].

CCCP inhibits and even diminishes the phosphorylation of STING, impairing the mitochondrial membrane potential to disrupt the interaction between TBK-1 and STING, eventually attenuating IFN-I-mediated inflammatory responses [114].

Using cryoelectron microscopy (cryo-EM), the structures of STING filaments in both apo- and cGAMP-bound forms, the former of which binds two ER membranes to prevent TBK1 recruitment, resulting in the autoinhibition of STING. This study provides a comprehensive view of STING autoinhibition, contributing significantly to the existing knowledge of cGAS-STING signalling [116].

In summary, various inhibitors of cGAS-STING ­regulate cell physiological effects based on different mechanisms, among which are effective in inhibiting inflammation, attenuating autoimmune diseases in animals. However, these inhibitors have only been conducted in animal models without any data in the human body and the effective dose and toxicology still remain unclear. In the future, more researches, especially clinical studies, are appealing to disease treatments targeting cGAS-STING STING signalling.

## Conclusion and perspectives

The consequences resulting from activation of STING and its pathways play crucial roles in liver disorders. Although cGAS-STING signalling can help defend against external infections in the liver, overactive or aberrant activation of this pathway can be harmful. Further research is warranted to explore the modulation of STING expression for the treatment and prevention of liver disorders.

As discussed above, the cGAS-STING pathway has an inhibitory effect on HBV hepatitis, but a stimulating effect in ALD, NAFLD, and liver fibrosis. In addition, in liver injury and autoimmune hepatitis, the cGAS-STING signalling pathway aids disease progression.

More efforts are needed to solve the following issues. First, it is urgent to confirm clinical translation of laboratory experiments and to further understand the specific functions of cGAS-STING signalling during liver disease development. Second, reports have focused on finding reliable biomarkers and utilizing animal experiments and specific chemicals to observe cGAS-STING expression in mice and human samples. For example, notably, quantifying the expression of cGAMP and Ser366 STING phosphorylation are particular indicators of pathway activity and can be valuable clinical biomarkers that accurately represent cGAS-STING pathway involvement. The relationships between STING and autophagy, necroptosis, ferroptosis, and pyroptosis in various liver diseases should be extensively studied in the future research. In addition, it is important to comprehensively investigate the mechanisms of the STING pathway in liver inflammatory diseases and explore techniques to control PCD-associated genes.

Although the cGAS-STING pathway is significant in liver inflammatory diseases, it is crucial to note that the intricacy of liver inflammation. Utilizing particular combinations of medications in the cGAS-STING pathway could be a potential approach to treat liver inflammation. Moreover, more studies are required to apply the current research results to medications that inhibit or stimulate the cGAS-STING pathway in liver inflammatory diseases.

## Data Availability

Data sharing is not applicable to this article as no new data were created or analysed in this study.

## References

[1] Asrani SK, Devarbhavi H, Eaton J, et al. Burden of liver diseases in the world. J Hepatol. 2019;70(1):151–171. doi: 10.1016/j.jhep.2018.09.014. 30266282

[2] Halpern KB, Shenhav R, Matcovitch-Natan O, et al. Single-cell spatial reconstruction reveals global division of labour in the mammalian liver. Nature. 2017;542(7641):352–356. doi: 10.1038/nature21065. 28166538 PMC5321580

[3] Trefts E, Gannon M, Wasserman DH. The liver. Curr Biol. 2017;27(21):R1147–R1151. doi: 10.1016/j.cub.2017.09.019. 29112863 PMC5897118

[4] Barbier L, Ferhat M, Salamé E, et al. Interleukin-1 family cytokines: keystones in liver inflammatory diseases. Front Immunol. 2019;10:2014. doi: 10.3389/fimmu.2019.02014. 31507607 PMC6718562

[5] Gong J, Tu W, Liu J, et al. Hepatocytes: a key role in liver inflammation. Front Immunol. 2022;13:1083780. doi: 10.3389/fimmu.2022.1083780. 36741394 PMC9890163

[6] Luedde T, Kaplowitz N, Schwabe RF. Cell death and cell death responses in liver disease: mechanisms and clinical relevance. Gastroenterology. 2014;147(4):765–783 e4. doi: 10.1053/j.gastro.2014.07.018. 25046161 PMC4531834

[7] Motwani M, Pesiridis S, Fitzgerald KA. DNA sensing by the cGAS-STING pathway in health and disease. Nat Rev Genet. 2019;20(11):657–674. doi: 10.1038/s41576-019-0151-1. 31358977

[8] Du S, Chen G, Yuan B, et al. DNA sensing and associated type 1 interferon signaling contributes to progression of radiation-induced liver injury. Cell Mol Immunol. 2021;18(7):1718–1728. doi: 10.1038/s41423-020-0395-x. 32203191 PMC8245603

[9] Decout A, Katz JD, Venkatraman S, et al. The cGAS-STING pathway as a therapeutic target in inflammatory diseases. Nat Rev Immunol. 2021;21(9):548–569. doi: 10.1038/s41577-021-00524-z. 33833439 PMC8029610

[10] Patel DJ, Yu Y, Xie W. cGAMP-activated cGAS-STING signaling: its bacterial origins and evolutionary adaptation by metazoans. Nat Struct Mol Biol. 2023;30(3):245–260. doi: 10.1038/s41594-023-00933-9. 36894694 PMC11749898

[11] Fang R, Jiang Q, Yu X, et al. Recent advances in the activation and regulation of the cGAS-STING pathway. Adv Immunol. 2022;156:55–102. doi: 10.1016/bs.ai.2022.09.003. 36410875

[12] Hopfner KP, Hornung V. Molecular mechanisms and cellular functions of cGAS-STING signalling. Nat Rev Mol Cell Biol. 2020;21(9):501–521. doi: 10.1038/s41580-020-0244-x. 32424334

[13] Zhang X, Bai XC, Chen ZJ. Structures and mechanisms in the cGAS-STING innate immunity pathway. Immunity. 2020;53(1):43–53. doi: 10.1016/j.immuni.2020.05.013. 32668227

[14] Wang C, Guan Y, Lv M, et al. Manganese increases the sensitivity of the cGAS-STING pathway for double-stranded DNA and is required for the host defense against DNA viruses. Immunity. 2018;48(4):675–687 e7. doi: 10.1016/j.immuni.2018.03.017. 29653696

[15] Zhao Z, Ma Z, Wang B, et al. Mn(2+) directly activates cGAS and structural analysis suggests Mn(2+) induces a noncanonical catalytic synthesis of 2’3’-cGAMP. Cell Rep. 2020;32(7):108053. doi: 10.1016/j.celrep.2020.108053. 32814054

[16] Kwon J, Bakhoum SF. The cytosolic DNA-sensing cGAS-STING pathway in cancer. Cancer Discov. 2020; 10(1):26–39. doi: 10.1158/2159-8290.CD-19-0761. 31852718 PMC7151642

[17] Benmerzoug S, Ryffel B, Togbe D, et al. Self-DNA sensing in lung inflammatory diseases. Trends Immunol. 2019;40(8):719–734. doi: 10.1016/j.it.2019.06.001. 31262653

[18] Ablasser A, Chen ZJ. cGAS in action: expanding roles in immunity and inflammation. Science. 2019;363(6431):eaat8657. doi: 10.1126/science.aat8657. 30846571

[19] Ablasser A, Hur S. Regulation of cGAS- and RLR-mediated immunity to nucleic acids. Nat Immunol. 2020; 21(1):17–29. doi: 10.1038/s41590-019-0556-1. 31819255

[20] Luo XJ, Li HG, Ma LQ, et al. Expression of STING is increased in liver tissues from patients with NAFLD and promotes macrophage-mediated hepatic inflammation and fibrosis in mice. Gastroenterology. 2018;155(6):1971–1984.e4. doi: 10.1053/j.gastro.2018.09.010. 30213555 PMC6279491

[21] Li A, Yi M, Qin S, et al. Activating cGAS-STING pathway for the optimal effect of cancer immunotherapy. J Hematol Oncol. 2019;2(1):35. doi: 10.1186/s13045-019-0721-x. PMC6444510 30935414

[22] Tannahill GM, Curtis AM, Adamik J, et al. Succinate is an inflammatory signal that induces IL-1beta through HIF-1alpha. Nature. 2013;496(7444):238–242. doi: 10.1038/nature11986. 23535595 PMC4031686

[23] Olagnier D, Brandtoft AM, Gunderstofte C, et al. Nrf2 negatively regulates STING indicating a link between antiviral sensing and metabolic reprogramming. Nat Commun. 2018;9(1):3506. doi: 10.1038/s41467-018-05861-7. 30158636 PMC6115435

[24] Murphy MP, O’Neill LAJ. Krebs cycle reimagined: the emerging roles of succinate and itaconate as signal transducers. Cell. 2018; 174(4):780–784. doi: 10.1016/j.cell.2018.07.030. 30096309

[25] Russo S, Kwiatkowski M, Govorukhina N, et al. Meta-inflammation and metabolic reprogramming of macrophages in diabetes and obesity: the importance of metabolites. Front Immunol. 2021;12:746151. doi: 10.3389/fimmu.2021.746151. 34804028 PMC8602812

[26] Viola A, Munari F, Sánchez-Rodríguez R, et al. The metabolic signature of macrophage responses. Front Immunol. 2019;10:1462. doi: 10.3389/fimmu.2019.01462. 31333642 PMC6618143

[27] Francque S, Szabo G, Abdelmalek MF, et al. Nonalcoholic steatohepatitis: the role of peroxisome proliferator-activated receptors. Nat Rev Gastroenterol Hepatol. 2021;18(1):24–39. doi: 10.1038/s41575-020-00366-5. 33093663

[28] Akhmetova K, Balasov M, Chesnokov I. Drosophila STING protein has a role in lipid metabolism. Elife. 2021;10;10:e67358. doi: 10.7554/eLife.67358. PMC8443252 34467853

[29] Hou YF, Wang ZM, Liu PY, et al. SMPDL3A is a cGAMP-degrading enzyme induced by LXR-mediated lipid metabolism to restrict cGAS-STING DNA sensing. Immunity. 2023;56(11):2492–2507.e10. doi: 10.1016/j.immuni.2023.10.001. 37890481

[30] Shin H, Chung H. SMPDL3A links cholesterol metabolism to the cGAS-STING pathway. Immunity. 2023; 56(11):2459–2461. doi: 10.1016/j.immuni.2023.10.015. 37967525 PMC11056274

[31] Vila IK, Chamma H, Steer A, et al. STING orchestrates the crosstalk between polyunsaturated fatty acid metabolism and inflammatory responses. Cell Metab. 2022;34(1):125–139 e8. doi: 10.1016/j.cmet.2021.12.007. 34986331 PMC8733004

[32] Gong J, Gao X, Ge S, et al. The role of cGAS-STING signalling in metabolic diseases: from signalling networks to targeted intervention. Int J Biol Sci. 2024;20(1):152–174. doi: 10.7150/ijbs.84890. 38164186 PMC10750282

[33] York AG, Williams KJ, Argus JP, et al. Limiting cholesterol biosynthetic flux spontaneously engages type I IFN signaling. Cell. 2015;163(7):1716–1729. doi: 10.1016/j.cell.2015.11.045. 26686653 PMC4783382

[34] Cruz VH, Arner EN, Wynne KW, et al. Loss of Tbk1 kinase activity protects mice from diet-induced metabolic dysfunction. Mol Metab. 2018;16:139–149. doi: 10.1016/j.molmet.2018.06.007. 29935921 PMC6157474

[35] Kumari M, Wang X, Lantier L, et al. IRF3 promotes adipose inflammation and insulin resistance and represses browning. J Clin Invest. 2016;126(8):2839–2854. doi: 10.1172/JCI86080. 27400129 PMC4966307

[36] Mao Y, Luo W, Zhang L, et al. STING-IRF3 triggers endothelial inflammation in response to free fatty acid-induced mitochondrial damage in diet-induced obesity. Arterioscler Thromb Vasc Biol. 2017;37(5):920–929. doi: 10.1161/ATVBAHA.117.309017. 28302626 PMC5408305

[37] Bertheloot D, Latz E, Franklin BS. Necroptosis, pyroptosis and apoptosis: an intricate game of cell death. Cell Mol Immunol. 2021;18(5):1106–1121. doi: 10.1038/s41423-020-00630-3. 33785842 PMC8008022

[38] Ohtani N. The roles and mechanisms of senescence-associated secretory phenotype (SASP): can it be controlled by senolysis? Inflamm Regen. 2022;242(1):11. 35365245 10.1186/s41232-022-00197-8 PMC8976373

[39] Coppé J-P, Patil CK, Rodier F, et al. Senescence-associated secretory phenotypes reveal cell-nonautonomous functions of oncogenic RAS and the p53 tumor suppressor. PLoS Biol. 2008;6(12):2853–2868. doi: 10.1371/journal.pbio.0060301. 19053174 PMC2592359

[40] Rodier F, Coppé JP, Patil CK, et al. Persistent DNA damage signalling triggers senescence-associated inflammatory cytokine secretion. Nat Cell Biol. 2009;11(8):973–979. doi: 10.1038/ncb1909. 19597488 PMC2743561

[41] Qi X, Zheng S, Ma M, et al. Curcumol suppresses CCF-mediated hepatocyte senescence through blocking LC3B-lamin B1 interaction in alcoholic fatty liver disease. Front Pharmacol. 2022;13:912825. doi: 10.3389/fphar.2022.912825. 35837283 PMC9273900

[42] Zhao DL, Gao YY, Su Y, et al. Oroxylin A regulates cGAS DNA hypermethylation induced by methionine metabolism to promote HSC senescence. Pharmacol Res. 2023;187:106590. doi: 10.1016/j.phrs.2022.106590. 36464146

[43] Sun Y, Weng J, Chen X, et al. Oroxylin A activates ferritinophagy to induce hepatic stellate cell senescence against hepatic fibrosis by regulating cGAS-STING pathway. Biomed Pharmacother. 2023;162:114653. doi: 10.1016/j.biopha.2023.114653. 37086511

[44] Sladitschek-Martens HL, Guarnieri A, Brumana G, et al. YAP/TAZ activity in stromal cells prevents ageing by controlling cGAS-STING. Nature. 2022;607(7920):790–798. doi: 10.1038/s41586-022-04924-6. 35768505 PMC7613988

[45] Nicholson DW, Thornberry NA. Caspases: killer proteases. Trends Biochem Sci. 1997;22(8):299–306. doi: 10.1016/s0968-0004(97)01085-2. 9270303

[46] Shen P, Han L, Chen G, et al. Emodin attenuates acetaminophen-induced hepatotoxicity via the cGAS-STING pathway. Inflammation. 2022;45(1):74–87. doi: 10.1007/s10753-021-01529-5. 34409550

[47] Wang Y, She S, Li W, et al. Inhibition of cGAS-STING pathway by stress granules after activation of M2 macrophages by human mesenchymal stem cells against drug induced liver injury. Mol Immunol. 2024;165:42–54. doi: 10.1016/j.molimm.2023.12.005. 38150981

[48] Wang X, Hu R, Song Z, et al. Sorafenib combined with STAT3 knockdown triggers ER stress-induced HCC apoptosis and cGAS-STING-mediated anti-tumor immunity. Cancer Lett. 2022;547:215880. doi: 10.1016/j.canlet.2022.215880. 35981569

[49] Liu Z, Wang M, Wang X, et al. XBP1 deficiency promotes hepatocyte pyroptosis by impairing mitophagy to activate mtDNA-cGAS-STING signaling in macrophages during acute liver injury. Redox Biol. 2022;52:102305. doi: 10.1016/j.redox.2022.102305. 35367811 PMC8971356

[50] Zhang X, Du J, Huo S, et al. Hexafluoropropylene oxide trimer acid causes fibrosis in mice liver via mitochondrial ROS/cGAS-STING/NLRP3-mediated pyroptosis. Food Chem Toxicol. 2023;174:113706. doi: 10.1016/j.fct.2023.113706. 36871880

[51] Jiang X, Stockwell BR, Conrad M. Ferroptosis: mechanisms, biology and role in disease. Nat Rev Mol Cell Biol. 2021;22(4):266–282. doi: 10.1038/s41580-020-00324-8. 33495651 PMC8142022

[52] Su W, Gao W, Zhang R, et al. TAK1 deficiency promotes liver injury and tumorigenesis via ferroptosis and macrophage cGAS-STING signalling. JHEP Rep. 2023;5(5):100695. doi: 10.1016/j.jhepr.2023.100695. 36968217 PMC10033999

[53] Li Y, Yu P, Fu W, et al. Ginsenoside Rd inhibited ferroptosis to alleviate CCl(4)-induced acute liver injury in mice via cGAS/STING pathway. Am J Chin Med. 2023;51(1):91–105. doi: 10.1142/S0192415X23500064. 36437551

[54] Zhang S, Kang L, Dai X, et al. Manganese induces tumor cell ferroptosis through type-I IFN dependent inhibition of mitochondrial dihydroorotate dehydrogenase. Free Radic Biol Med. 2022;193(Pt 1):202–212. doi: 10.1016/j.freeradbiomed.2022.10.004. 36228830

[55] Qiu S, Zhong X, Meng X, et al. Mitochondria-localized cGAS suppresses ferroptosis to promote cancer progression. Cell Res. 2023;33(4):299–311. doi: 10.1038/s41422-023-00788-1. 36864172 PMC10066369

[56] Megahed FAK, Zhou XL, Sun PN. The interactions between HBV and the innate immunity of hepatocytes. Viruses-Basel. 2020;12(3):285. doi: 10.3390/v12030285. PMC7150781 32151000

[57] Jiang S, Xia N, Luo J, et al. The porcine cyclic GMP-AMP synthase-STING pathway exerts an unusual antiviral function independent of interferon and autophagy. J Virol. 2022; 96(23):e0147622. doi: 10.1128/jvi.01476-22. 36377876 PMC9749457

[58] Lv L, Chai L, Wang J, et al. Selenoprotein K enhances STING oligomerization to facilitate antiviral response. PLoS Pathog. 2023;19(4):e1011314. doi: 10.1371/journal.ppat.1011314. 37023217 PMC10112805

[59] Yu P, Miao Z, Li Y, et al. cGAS-STING effectively restricts murine norovirus infection but antagonizes the antiviral action of N-terminus of RIG-I in mouse macrophages. Gut Microbes. 2021;13(1):1959839. doi: 10.1080/19490976.2021.1959839. 34347572 PMC8344765

[60] He J, Hao R, Liu D, et al. Inhibition of hepatitis B virus replication by activation of the cGAS-STING pathway. J Gen Virol. 2016;97(12):3368–3378. doi: 10.1099/jgv.0.000647. 27902332

[61] Dansako H, Ueda Y, Okumura N, et al. The cyclic GMP-AMP synthetase-STING signaling pathway is required for both the innate immune response against HBV and the suppression of HBV assembly. Febs J. 2016;3(1):144–156. doi: 10.1111/febs.13563. 26471009

[62] Chen H, Jiang L, Chen S, et al. HBx inhibits DNA sensing signaling pathway via ubiquitination and autophagy of cGAS. Virol J. 2022;19(1):55. doi: 10.1186/s12985-022-01785-3. 35346247 PMC8962493

[63] Zhao J, Xu G, Hou X, et al. Schisandrin C enhances cGAS-STING pathway activation and inhibits HBV ­replication. J Ethnopharmacol. 2023;311:116427. doi: 10.1016/j.jep.2023.116427. 37001770

[64] Shu D, Cheng L, Yuan K, et al. RVX-208, an inducer of Apolipoprotein A-I, inhibits the particle production of hepatitis B virus through activation of cGAS-STING pathway. Antivir Ther. 2023;28(6):13596535231219639. 38037795 10.1177/13596535231219639

[65] Yu Z, Tong L, Ma C, et al. The UAF1-USP1 deubiquitinase complex stabilizes cGAS and facilitates antiviral responses. J Immunol. 2024; 212(2):295–301. doi: 10.4049/jimmunol.2200462. 38054892

[66] Mihm S. Danger-associated molecular patterns (DAMPs): molecular triggers for sterile inflammation in the liver. Int J Mol Sci. 2018;19(10):3104. doi: 10.3390/ijms19103104. 30309020 PMC6213769

[67] Allison R, Guraka A, Shawa IT, et al. Drug induced liver injury - a 2023 update. J Toxicol Environ Health B Crit Rev. 2023;26(8):442–467. doi: 10.1080/10937404.2023.2261848. 37786264

[68] Bunchorntavakul C, Reddy KR. Acetaminophen (APAP or N-acetyl-p-aminophenol) and acute liver failure. Clin Liver Dis. 2018;22(2):325–346. doi: 10.1016/j.cld.2018.01.007. 29605069

[69] Msolli MA, Sekma A, Toumia M, et al. Acetaminophen, nonsteroidal anti-inflammatory drugs, or combination of both analgesics in acute posttrauma pain: a randomized controlled trial. Acad Emerg Med. 2021;28(2):155–163. doi: 10.1111/acem.14169. 33145862

[70] Zhong Y, Chen Y, Pan Z, et al. Ginsenoside Rc, as an FXR activator, alleviates acetaminophen-induced hepatotoxicity via relieving inflammation and oxidative stress. Front Pharmacol. 2022;13:1027731. doi: 10.3389/fphar.2022.1027731. 36278209 PMC9585238

[71] Araujo AM, Antunes MM, Mattos MS, et al. Liver immune cells release type 1 interferon due to DNA sensing and amplify liver injury from acetaminophen overdose. Cells. 2018;7(8):88. doi: 10.3390/cells7080088. 30060463 PMC6115735

[72] Zhong W, Rao Z, Xu J, et al. Defective mitophagy in aged macrophages promotes mitochondrial DNA cytosolic leakage to activate STING signaling during liver sterile inflammation. Aging Cell. 2022;21(6):e13622. doi: 10.1111/acel.13622. 35599014 PMC9197407

[73] Zhong W, Rao Z, Rao J, et al. Aging aggravated liver ischemia and reperfusion injury by promoting STING-mediated NLRP3 activation in macrophages. Aging Cell. 2020;19(8):e13186. doi: 10.1111/acel.13186. 32666684 PMC7431827

[74] Wu XY, Chen YJ, Liu CA, et al. STING induces liver ischemia-reperfusion injury by promoting calcium-dependent caspase 1-GSDMD processing in macrophages. Oxid Med Cell Longev. 2022;2022:8123157. doi: 10.1155/2022/8123157. 35281468 PMC8906939

[75] Shen A, Zheng D, Luo Y, et al. MicroRNA-24-3p alleviates hepatic ischemia and reperfusion injury in mice through the repression of STING signaling. Biochem Biophys Res Commun. 2020;522(1):47–52. doi: 10.1016/j.bbrc.2019.10.182. 31735332

[76] Kong E, Zhang Y, Geng X, et al. Inhibition of Sirt3 activates the cGAS-STING pathway to aggravate hepatocyte damage in hepatic ischemia-reperfusion injury mice. Int Immunopharmacol. 2024;128:111474. doi: 10.1016/j.intimp.2023.111474. 38185036

[77] Koay EJ, Owen D, Das P. Radiation-induced liver disease and modern radiotherapy. Semin Radiat Oncol. 2018;28(4):321–331. doi: 10.1016/j.semradonc.2018.06.007. 30309642 PMC6402843

[78] Li J, Lu Y, Lin G. Blocking cGAS/STING signaling protects against sepsis-associated acute liver injury. Int Immunopharmacol. 2022;113(Pt A):109276. doi: 10.1016/j.intimp.2022.109276. 36252490

[79] Liu J, Tang M, Li Q, et al. ATG2B upregulated in LPS-stimulated BMSCs-derived exosomes attenuates septic liver injury by inhibiting macrophage STING signaling. Int Immunopharmacol. 2023;117:109931. doi: 10.1016/j.intimp.2023.109931. 36857936

[80] Wang X, Rao H, Zhao J, et al. STING expression in monocyte-derived macrophages is associated with the progression of liver inflammation and fibrosis in patients with nonalcoholic fatty liver disease. Lab Invest. 2020;100(4):542–552. doi: 10.1038/s41374-019-0342-6. 31745210

[81] Donne R, Saroul-Ainama M, Cordier P, et al. Replication stress triggered by nucleotide pool imbalance drives DNA damage and cGAS-STING pathway activation in NAFLD. Dev Cell. 2022;57(14):1728–1741 e6. doi: 10.1016/j.devcel.2022.06.003. 35768000

[82] Luo ZL, Ji YD, Zhang DH, et al. Microbial DNA enrichment promotes liver steatosis and fibrosis in the course of non-alcoholic steatohepatitis. Acta Physiol. 2022;235(3):e13827. doi: 10.1111/apha.13827. PMC9335517 35500155

[83] Powell EE, Wong VW, Rinella M. Non-alcoholic fatty liver disease. Lancet. 2021;97(10290):2212–2224. doi: 10.1016/S0140-6736(20)32511-3. 33894145

[84] Fuchs CD, Radun R, Dixon ED, et al. Hepatocyte-specific deletion of adipose triglyceride lipase (adipose ­triglyceride lipase/patatin-like phospholipase domain containing 2) ameliorates dietary induced steatohepatitis in mice. Hepatology. 2022;75(1):125–139. doi: 10.1002/hep.32112. 34387896

[85] Cho CS, Park HW, Ho A, et al. Lipotoxicity induces hepatic protein inclusions through TANK binding kinase 1-mediated p62/sequestosome 1 phosphorylation. Hepatology. 2018;68(4):1331–1346. doi: 10.1002/hep.29742. 29251796 PMC6005718

[86] Luo W, Xu G, Song Z, et al. Licorice extract inhibits the cGAS-STING pathway and protects against non-alcoholic steatohepatitis. Front Pharmacol. 2023;14:1160445. doi: 10.3389/fphar.2023.1160445. 37081966 PMC10111149

[87] Caputo F, Domenicali M, Bernardi M. Diagnosis and treatment of alcohol use disorder in patients with end-stage alcoholic liver disease. Hepatology. 2019; 70(1):410–417. doi: 10.1002/hep.30358. 30471136

[88] Wen B, Zhang C, Zhou J, et al. Targeted treatment of alcoholic liver disease based on inflammatory signalling pathways. Pharmacol Ther. 2021;222:107752. doi: 10.1016/j.pharmthera.2020.107752. 33253739

[89] Ma X, Chen A, Melo L, et al. Loss of hepatic DRP1 exacerbates alcoholic hepatitis by inducing megamitochondria and mitochondrial maladaptation. Hepatology. 2023;77(1):159–175. doi: 10.1002/hep.32604. 35698731 PMC9744966

[90] Wang Q, Bu Q, Liu M, et al. XBP1-mediated activation of the STING signalling pathway in macrophages contributes to liver fibrosis progression. JHEP Rep. 2022; 4(11):100555. doi: 10.1016/j.jhepr.2022.100555. 36185574 PMC9520276

[91] Yong H, Wang S, Song FY. Activation of cGAS/STING pathway upon TDP-43-mediated mitochondrial injury may be involved in the pathogenesis of liver fibrosis. Liver Int. 2021;41(8):1969–1971. doi: 10.1111/liv.14895. 33830629

[92] Shan SL, Liu ZD, Wang S, et al. Mitochondrial oxidative stress regulates LonP1-TDP-43 pathway and rises mitochondrial damage in carbon tetrachloride-induced liver fibrosis. Ecotox Environ Safe. 2023; 264:115409. doi: 10.1016/j.ecoenv.2023.115409. 37647804

[93] Xiao Y, Zhao C, Tai Y, et al. STING mediates hepatocyte pyroptosis in liver fibrosis by Epigenetically activating the NLRP3 inflammasome. Redox Biol. 2023;62:102691. doi: 10.1016/j.redox.2023.102691. 37018971 PMC10106968

[94] Luo S, Luo R, Lu H, et al. Activation of cGAS-STING signaling pathway promotes liver fibrosis and hepatic sinusoidal microthrombosis. Int Immunopharmacol. 2023;125(Pt B):111132. doi: 10.1016/j.intimp.2023.111132. 37951190

[95] Gu L, Zhao C, Wang Y, et al. Senescence of hepatic stellate cells by specific delivery of manganese for limiting liver fibrosis. Nano Lett. 2024;24(4):1062–1073. 2024/01/02. doi: 10.1021/acs.nanolett.3c03689. 38164915 PMC10836362

[96] Mullard A. Biotechs step on cGAS for autoimmune diseases. Nat Rev Drug Discov. 2023;22(12):939–941. doi: 10.1038/d41573-023-00185-8. 37949966

[97] Hou P, Lin Y, Li Z, et al. Autophagy receptor CCDC50 tunes the STING-mediated interferon response in viral infections and autoimmune diseases. Cell Mol Immunol. 2021;18(10):2358–2371. doi: 10.1038/s41423-021-00758-w. 34453126 PMC8484562

[98] Mieli-Vergani G, Vergani D, Czaja AJ, et al. Autoimmune hepatitis. Nat Rev Dis Primers. 2018;24(1):18017. doi: 10.1038/nrdp.2018.17. 29644994

[99] Saimaier K, Han S, Lv J, et al. Manganese exacerbates ConA-induced liver inflammation via the cGAS-STING signaling pathway. Inflammation. 2024;47(1):333–345. doi: 10.1007/s10753-023-01912-4. 37805951

[100] Hall J, Brault A, Vincent F, et al. Discovery of PF-06928215 as a high affinity inhibitor of cGAS enabled by a novel fluorescence polarization assay. PLoS One. 2017;12(9):e0184843. doi: 10.1371/journal.pone.0184843. 28934246 PMC5608272

[101] Vincent J, Adura C, Gao P, et al. Small molecule inhibition of cGAS reduces interferon expression in primary macrophages from autoimmune mice (vol 8, 750, 2017). Nat Commun. 2017;8(1):750. doi: 10.1038/s41467-017-01770-3. PMC5622107 28963528

[102] Lama L, Adura C, Xie W, et al. Development of human cGAS-specific small-molecule inhibitors for repression of dsDNA-triggered interferon expression. Nat Commun. 2019;10(1):2261. doi: 10.1038/s41467-019-08620-4. 31113940 PMC6529454

[103] Zhao WF, Xiong MY, Yuan XJ, et al. In silico screening-based discovery of novel inhibitors of human cyclic GMP-AMP synthase: a cross-validation study of molecular docking and experimental testing. J Chem Inf Model. 2020;60(6):3265–3276. doi: 10.1021/acs.jcim.0c00171. 32459092

[104] Wang M, Sooreshjani MA, Mikek C, et al. Suramin potently inhibits cGAMP synthase, cGAS, in THP1 cells to modulate IFN-beta levels. Future Med Chem. 2018; 10(11):1301–1317. doi: 10.4155/fmc-2017-0322. 29558821

[105] An J, Minie M, Sasaki T, et al. Antimalarial drugs as immune modulators: new mechanisms for old drugs. Annu Rev Med. 2017;68(1):317–330. doi: 10.1146/annurev-med-043015-123453. 27813878

[106] Siu T, Altman MD, Baltus GA, et al. Discovery of a novel cGAMP competitive ligand of the inactive form of STING. ACS Med Chem Lett. 2019;10(1):92–97. doi: 10.1021/acsmedchemlett.8b00466. 30655953 PMC6331172

[107] Steinhagen F, Zillinger T, Peukert K, et al. Suppressive oligodeoxynucleotides containing TTAGGG motifs inhibit cGAS activation in human monocytes. Eur J Immunol. 2018;48(4):605–611. doi: 10.1002/eji.201747338. 29215161 PMC6386451

[108] Li J, Xiong M, Liu J, et al. Discovery of novel cGAS inhibitors based on natural flavonoids. Bioorg Chem. 2023;140:106802. doi: 10.1016/j.bioorg.2023.106802. 37666112

[109] Li S, Hong Z, Wang Z, et al. The cyclopeptide astin C specifically inhibits the innate immune CDN sensor STING. Cell Rep. 2018;25(12):3405–3421 e7. doi: 10.1016/j.celrep.2018.11.097. 30566866

[110] Tian X, Xu F, Zhu Q, et al. Medicinal chemistry perspective on cGAS-STING signaling pathway with small molecule inhibitors. Eur J Med Chem. 2022;244:114791. doi: 10.1016/j.ejmech.2022.114791. 36206657

[111] Haag SM, Gulen MF, Reymond L, et al. Targeting STING with covalent small-molecule inhibitors. Nature. 2018;559(7713):269–273. doi: 10.1038/s41586-018-0287-8. 29973723

[112] Mukai K, Konno H, Akiba T, et al. Activation of STING requires palmitoylation at the Golgi. Nat Commun. 2016;7(1):11932. doi: 10.1038/ncomms11932. 27324217 PMC4919521

[113] Hansen AL, Buchan GJ, Rühl M, et al. Nitro-fatty acids are formed in response to virus infection and are ­potent inhibitors of STING palmitoylation and signaling. Proc Natl Acad Sci U S A. 2018;115(33):E7768–E7775. 30061387 10.1073/pnas.1806239115 PMC6099880

[114] Kwon D, Park E, Sesaki H, et al. Carbonyl cyanide 3-chlorophenylhydrazone (CCCP) suppresses STING-mediated DNA sensing pathway through inducing ­mitochondrial fission. Biochem Biophys Res Commun. 2017;493(1):737–743. doi: 10.1016/j.bbrc.2017.08.121. 28859978

[115] An J, Woodward JJ, Lai W, et al. Inhibition of cyclic GMP-AMP synthase using a novel antimalarial drug derivative in trex1-deficient mice. Arthritis Rheumatol. 2018;70(11):1807–1819. doi: 10.1002/art.40559. 29781188

[116] Liu S, Yang B, Hou Y, et al. The mechanism of STING autoinhibition and activation. Mol Cell. 2023;83(9):1502–1518 e10. doi: 10.1016/j.molcel.2023.03.029. 37086726

